# Aging 4.0? Rethinking the ethical framing of technology-assisted eldercare

**DOI:** 10.1007/s40656-021-00447-x

**Published:** 2021-08-03

**Authors:** Silke Schicktanz, Mark Schweda

**Affiliations:** 1 Department of Medical Ethics and History of Medicine, University Medical Center Göttingen, Humboldt Allee 36, 37073 Göttingen, Germany; 2 Department of Health Services Research, School of Medicine and Health Sciences, Carl von Ossietzky University of Oldenburg, Ammerländer Heerstr. 114-118, 26111 Oldenburg, Germany

**Keywords:** Ethics, Aging, Technology assisted care, Empowerment, Privacy, Liability, Justice

## Abstract

Technological approaches are increasingly discussed as a solution for the provision of support in activities of daily living as well as in medical and nursing care for older people. The development and implementation of such assistive technologies for eldercare raise manifold ethical, legal, and social questions. The discussion of these questions is influenced by theoretical perspectives and approaches from medical and nursing ethics, especially the principlist framework of autonomy, non-maleficence, beneficence, and justice. Tying in with previous criticism, the present contribution is taking these principles as a starting point and as a frame of reference to be critically re-examined. It thus aims to outline how existing ethical frameworks need to be extended or reconsidered to capture the ethical issues posed by technological developments regarding care for older people. In a first step, we provide a brief overview of assistive technologies in eldercare according to their purposes and functions. In the next step, we discuss how the questions and problems raised by new technologies in eldercare call for an expansion, re-interpretation, and revision of the principlist framework. We underline that the inclusion of ethical perspectives from engineering and computer science as well as a closer consideration of socio-political dimensions and fundamental anthropological and praxeological questions are needed.

## Introduction

Due to demographic aging, the change of traditional familial care arrangements, and the shortage of professional care workers, technological approaches are increasingly discussed as a solution for ensuring the provision of support in activities of daily living as well as in medical and nursing care for older people. Such assistive technologies comprise a broad spectrum of devices and systems, such as apps to diagnose current health status, various forms of telemedicine, robotics to support independent living as well as formal and informal care, and intelligent sensor- and monitoring systems for ambient assisted living, for example, in smart homes.

Of course, the development and implementation of such technological systems in a vulnerable population like frail and dependent older people and in a sensitive area like eldercare raise manifold ethical, legal, and social questions. The discussion of ethical, legal and social implications of technology-assisted eldercare has been rapidly evolving throughout the last decade and has produced preliminary explorations and outlines of ethical problems (Anderson & Anderson, 2008; Sharkey & Sharkey, 2012), more systematic approaches and models of ethical analysis and evaluation (e.g., Ammicht Quinn et al., 2015; Krenn, 2015; Manzeschke et al., 2015) and also a few comprehensive reviews of the debate itself (e.g., Hofmann, 2013; Vandemeulebroucke et al., 2018; Zwijsen et al., 2011). However, as Ieanca et al. (2018) conclude in their impressive descriptive review of over 500 articles addressing – among other things – the ‘ethical design’ of such technologies, the list of ethical topics raised appears rather limited. The majority of such technologies were developed without any explicit ethical consideration at all. If ethical aspects were considered, it was mainly the following six (in this order of prevalence): first, autonomy, then non-maleficence, beneficence, justice, interdependence, and, most rarely, privacy. This list indicates a lasting influence of ethical perspectives and approaches originally formulated in the context of medicine and healthcare.

Indeed, the influence of the principlist framework of biomedical ethics in the ethical discussion of technology-assisted eldercare has been noted repeatedly (Schmietow, 2020; Vandemeulebroucke et al., 2018). Many analyses of ethical problems in technology-assisted eldercare explicitly apply the four principles of autonomy, non-maleficence, beneficence, and justice (Preuß & Legal, 2017; Ienca et al., 2016, 2018; Körtner, 2016; Sorell & Draper, 2014; Feil-Seifer & Mataric, 2011). Some concentrate on only one or two central ethical principles, with a special focus resting on autonomy (Sharkey & Sharkey, 2012; Sparrow & Sparrow, 2006). Others start from the principlist framework and expand or differentiate the set of principles in order to adapt it to the specific technological context, for example, highlighting aspects of privacy, safety or self-conception (Ammicht Quinn et al., 2015; Manzeschke et al., 2015). Of course, there are also proposals that go beyond this framework, such as Vallor’s (2011) use of the capability approach or van Wynsberghe’s (2013) care-centered approach for the ethical design of care robots that focuses on care-ethical principles such as attentiveness, responsibility, competence, and responsiveness. All in all, however, the principlist approach frequently provides the normative foundation and starting point of ethical analysis and evaluation in technology-assisted eldercare.

Several commentators have pointed out that the area of technology-assisted eldercare requires a shift or expansion of this medical ethical perspective to comprise and unfold the techno-ethical dimension (Brown & Adams, 2007; Silvers, 2010). This includes the perspective that users are not mainly seen as ‘patients’ placed in a carer-patient- or doctor-patient-relationship, but as citizens with particular rights. Following this line of thought, we take the common principles of biomedical ethics – autonomy, care (comprising non-maleficence as well as beneficence), and justice – as a starting point and as frame of reference to be critically re-examined. The article thus offers a programmatic outline of how existing ethical frameworks can capture the new ethical issues posed by technological developments regarding care for older people. In a first step, we provide a brief overview of assistive technologies in eldercare according to their different purposes and functions. In the next step, we discuss how the questions and problems raised by new technologies in eldercare require an expansion, re-interpretation and – at least in some points – revision of the principlist framework of autonomy, care, and justice. We point out that the inclusion of ethical perspectives from engineering and computer science as well as a closer consideration of socio-political dimensions and fundamental anthropological and praxeological questions are needed.

## Background: assistive technologies in eldercare

Over the last decade, the scope and variety of assistive technologies for eldercare have virtually exploded. Self-tracking devices and wearable sensors allow the continuous collection and analysis of physiological parameters and movement data, and thus a close control of body functions and behavior patterns (Wang et al., 2017). Monitoring technologies surveil regular patterns of everyday life activities and initiate alarms in case of deviations from normal standards (Wagner et al., 2012). Coupled with learning algorithms and robotic devices, they form systems that can adapt to new contexts and intervene in given situations to support everyday life or care processes (Martinez-Martin & del Pobil, 2018). Larger connected networks of such systems constitute the technological infrastructure of ambient assisted living for older people (Liu et al., 2016). These technologies represent a new generation of assistive systems based on digitalization, networks, and flexibility. In analogy to discussions about the Fourth Industrial Revolution (Lasi et al., 2014), it could be labeled as the 4.0 version of technology-assisted eldercare – the latest ideal of technological development in this field. Of course, many approaches are still in their infancy. Yet, this early stage of technological development is an advantage for providing guiding ethical reflection.

All these technologies have in common that they involve some form of human-machine-interaction. Thus, they are best described as human-cyber-physical systems (Liu & Wang, 2020) or socio-technical systems. Such systems are defined by complex feedback loops, control functions, and communication. Therefore, their development and implementation require not only technological expertise from fields like computer science and engineering, but also psychological, sociological, and – in the case of eldercare – also gerontological knowledge (Schulz et al., 2015). Furthermore, especially in a vulnerable population like more or less frail and dependent older people and in a particularly sensitive context like eldercare, such systems have to be considered as safety-critical: their failure can result in serious damages to the life, health, and well-being of human beings (Miller et al., 2002).

With regard to their purpose and function, different types of assistive technologies for eldercare can be distinguished: wearables, intelligent life assistants, robotics, and smart homes (Byrne et al., 2018). *Wearables* are sensor-based technical devices that can be attached directly to the human body or inserted into garments. They can be used to control vital parameters, physiological functions, and behavioral patterns over longer periods of time and report irregular events or deviant developments. Possible fields of application comprise health-monitoring, fall detection, and the surveillance of activities of everyday life (Stavropoulos et al., 2020). For example, there are wrist-worn wearables that continuously measure physiological parameters such as pulse, blood pressure, and glucose levels and initiate an alarm in case of deviance from individual standard values (ibid.).

*Intelligent life assistants* are smart tracking technologies and positioning systems that help to orient, locate, and monitor older people in their own homes or their larger outdoor environments (Byrne et al., 2018). They comprise fall detection devices involving ambient depth cameras or infrared sensors to monitor an individual’s physical movements and location in space, or floor pressure sensors that can detect longer periods of immobility. Another field of application is the support of spatial orientation and navigation, for example, through GPS or radar technology, as well as the prevention of wandering with the help of geofencing systems, especially for older people with cognitive impairments. Finally, electronic home control systems are sensor-based smart technologies that monitor behavioral patterns and offer assistance in performing activities of daily life such as cooking, clothing, or personal hygiene by providing visual or acoustic prompts and information, or daily routine reminders, for example, for meals, exercise, or medication (ibid.).

*Smart robotic systems* can provide direct assistance by physically intervening in and helping with activities of daily living through a combination of sensor, processing, and actuator technologies. They comprise intelligent physical movement aids as well as service and companion robots for specific care or household tasks (Byrne et al., 2018). Intelligent physical movement aids are robotic technologies that support bodily movements of frail or disabled older people, for example, by means of gripping devices, smart walkers, or even external robotic limbs adapting movements to normal levels. Some robotic systems provide comprehensive physical support of activities of daily living, healthcare, and housekeeping. A special subclass includes socially assistive robots (SAR). These service and companion robots provide emotional and social assistance, for example, by offering stimuli for and support with communication, play and entertainment, or leisure activities (Kachouie et al., 2014). A prominent example is PARO, an interactive robotic baby seal designed to trigger positive emotions and stimulate social interactions, especially in people with dementia (http://www.parorobots.com/).

Lastly, *smart home technologies* are comprehensive smart infrastructures systematically interconnecting and combining the technological approaches and functions of sensors and wearables, intelligent life assistants, and robotic systems (Byrne et al., 2018). Based on interlinked platforms, they are aimed at automatically monitoring and supporting a whole variety of aspects of everyday life and thus helping older people to maintain a largely independent form of living in their own household. For example, smart homes connect functions of health and behavior monitoring, assistance with physical mobility, activities of daily living, the regulation of household functions and services, as well as tele-presence, communication, and social inclusion (Morris et al., 2013). These technological functions of smart homes correspond to the vision of ambient assisted living (AAL) embedded in a comprehensive technological environment which enables older people to lead an independent and self-determined life and stay in their own homes and communities (ibid.).

Looking at the general structure and functioning of the human-machine-interaction, these groups of assistive technologies for eldercare can be further classified according to different levels of automation that define the respective roles and abilities of both participants in the socio-technical system (Kaber, 2018). The basic level is marked by largely passive technological devices that human users utilize as mere tools, for example, a bathtub lift that is operated by a remote control. At the other end of the spectrum, the highest level comprises fully autonomous technological systems that are able to make all relevant decisions on their own and thus do not require any active human user intervention. Between these two endpoints, different forms of “co-intelligent” systems (Schweda et al., 2020) constitute a broad middle-ground. On the one hand, these systems employ methods from artificial intelligence (AI) and machine learning (ML) and thus show a certain capacity of self-regulation. On the other hand, they still rely on varying degrees of human decision-making and inputs to be able to function. In this sense, they combine human and artificial intelligence. This distinguishes co-intelligent technologies from both traditional assistive devices without smart computing functions and the vision of fully automated assistive systems, such as intelligent robots.

## Rethinking the ethics of eldercare in the age of technological assistance

As several commentators have pointed out, the ethical evaluation of these new emerging technologies that are starting to revolutionize the daily lives of and the care for older people transcends the medical perspective that still dominates many debates on ethics and aging. With engineering and computer science as the leading disciplines in this new technological development, new scientific and technological aspects as well as different professions and, with them, a different professional ethos come into play (Brown & Adams, 2007; Silvers, 2010). Therefore, it is necessary to expand and modify ethical frameworks to address the specific moral issues of technology-assisted eldercare. Starting from the basic principlist framework of biomedical ethics defined by autonomy, non-maleficence and beneficence (here subsumed under the principle of care), and justice, we will critically examine and expand the corresponding ethical perspectives. As we want to argue, technology ethics cannot substitute healthcare ethics. What we need is rather an expansion and integration of various lines of ethical reasoning.

### Specifying respect for autonomy: the importance of empowerment and privacy

In biomedical ethics, the idea of patient autonomy basically addresses the right to self-determination about one’s own medical treatment. The concrete practical manifestation of this right is the procedure of informed consent: Based on adequate information about the planned intervention, the patient makes a voluntary and deliberate decision that the doctor has to respect. In the context of technology-assisted eldercare, our basic understanding of respect towards a patient’s self-determination is substantially expanded by pertinent concepts of empowerment and privacy. Taking these into account can help to criticize simplistic or reductionist understandings of autonomy.

#### Empowerment: establishing structural preconditions for practicing self-determination

The term ‘empowerment’ is prominently used in the context of health promotion, self-management for chronic illness, or social work (McWilliam, 2009; WHO, 1998). As a concept, it was originally developed in the context of emancipatory pedagogy, social psychology, mental health, and health promotion (Chiapperino & Tengland, 2015). The core idea of empowerment can be defined by three basic claims: support of independence, political participation, and a shift in pre-existing power relations (e.g., Aujoulat et al., 2008; Castro et al., 2016; Feste & Anderson, 1995; Kayser et al., 2019). This theoretical basis is reflected in a recent empirical study by McConnell et al. (2019) which focuses on the views of people with dementia (PWD) and finds that these have the following understanding of empowerment: “A confidence building process whereby PWD are respected, have a voice and are heard, are involved in making decisions about their lives and have the opportunity to create change through access to appropriate resources” (ibid., p. 2).

In this sense, empowerment goes beyond the traditional understanding of self-determination. Instead of presuming a patient who can choose between alternatives, it starts from a broader ontological assumption of vulnerabilities (Bergmann & Frewer, 2018). As Anita Silvers (2010) pointed out, assistive technologies in care, especially when guided by medical approaches, risk to discriminate against persons with disabilities if they presume “the aim [was] to restore a disabled person as closely as possible to normality” (ibid., p. 10). Thus, from the perspective of an ethics of technology, it can be more conducive in terms of empowerment to support diverse functionality over ideas of ‘normal’ functioning that reproduce a logic of deficiency (Ladner, 2010). Cook et al. (2010) provide practical examples of how the involvement of people with disability in the design and implementation of technology can avoid such pitfalls of assistive technology development.

Empowerment theory further stresses the relevance of social relations for exercising individual self-determination and sensitizes us to the role that (asymmetrical) power relations play in its execution (Rose, 1990) – especially as these seem pervasive in healthcare settings. While patients (particularly more or less frail and older people) are usually in a comparatively powerless position, other agents, such as healthcare providers, health bureaucrats, nursing professionals, and doctors often possess more power. Empowerment is therefore instructive when discussing technologies for older people and can help to reflect on explicit and implicit assumptions of power relations with regard to aging and technology development. Substantial empowerment should address conditions on the structural level of organizations and the social system rather than impose the burden of agency solely on the individual level (Castro et al., 2016; Feste & Anderson, 1995). It is not enough to acknowledge the fact that the individual is socially embedded. Instead, we need to consider personal and structural dependencies that require continuous negotiation of (or even struggle over) how much a person accepts to be dominated by the expectations or directives of others, and how much one’s own agency relies on obedience or subordination to third parties.

Empowerment philosophy does not see power positions as fixed, but as malleable conditions. It also relies on a dual conception of power that distinguishes ‘power over’, that is, domination, from ‘power to’, as a factor or capacity that enables human agency (Clegg, 1989; Hauggard, 2002). The former can be described as instrumental power and the latter as facilitative power (McConnell et al., 2019). Instrumental power is enacted by elite structures, in the case of health care, for example, by service providers, politicians, or the technological industry. In this case, power is exercised at the expense of the technology-dependent users, e.g., by predetermining needs, costs, or accessibility. By contrast, facilitative power can be understood as a factor that enables an individual or a group of people to achieve their own goals. In this sense, empowerment is often understood as the normative ideal to bring facilitative power to those who have been dominated by others without creating new forms of domination. However, complex social webs and shifts in power relations can lead to new forms of power constellations with new rules, new meaning, or new membership (Clegg, 1989). These reflective, mutually related perspectives sensitize us to the fact that even attempts for more facilitative power can result in new forms of instrumental power. Hence, in the context of technological assistance, a leading question should always be how technologies contribute to established power asymmetries or to the reallocation of power. In general, to empower somebody should indicate a change of an asymmetric relationship into a more symmetrical one. For older people, empowerment can also mean to maintain existing capabilities and to avoid a drift towards even more asymmetric forms of dependence. In this sense, independence can mean aging and living in place and thus maintaining existing living conditions (Callahan, 2019).

To capture this emancipatory impetus of empowerment philosophy, we suggest to integrate a number of practical conditions for ethical considerations into the development of assistive technologies (adopted and refined from work by Feste and Anderson (1995), Small et al. (2013) and McConnell et al. (2019)): First, well-being is defined in a way that encourages people to identify their own values, needs, and goals, and does not impose external values. While earlier work on empowerment still defends the importance of a ‘healthy life’ (Feste & Anderson, 1995, p. 141), the context of eldercare requires a skeptical stance towards medically predefined ideals of ‘health’ that individuals must fulfill. Such a form of ‘healthism’ can lead to a problematic pathologization of aging.

Second, personal identity is perceived as processual; changes in self-images, e.g., by personal adaption of needs over the life course, have to be integrated (Schweda, 2017). Third, personal control over the decisions one sees as important is crucial, but it is acceptable if decisions are taken over by others in case of less relevant issues.

Fourth, factual knowledge or technical competence are only required at a level that seems sufficient for personal control. Fifth, innovations which support older people to enable others, e.g., by being of help or providing support for other persons, are valued. This counters the stereotype that older people are only dependent on support, and stresses the value of reciprocity for a good life.

Sixth, vulnerability is understood as a social constraint that can lead to stigmatization, that is, stereotyping, separation, status loss, and discrimination (Link & Phelan, 2001). In our case, the stigma of old age or age-associated conditions such as dementia need to be taken into account. Technological assistance must be designed in a way that does not increase such stigma. Empirical research helps to understand if and how assistive technologies can increase or reduce stigma. For example, some assistive technologies, such as walking aids, are not well accepted by older people because of fear of increased stigmatization (Yusif et al., 2016). The question is whether ‘smart’ technology that is small, invisible, and easy to control can help to reduce the stereotype that older people are ‘incompetent’ by giving them more personal control or independence. And finally, active participation of those affected at different levels of technology design is the most helpful tool to ensure that these complex, practical perspectives are sufficiently considered (Merkel & Kucharski, 2019).

In sum, the concept of empowerment calls for a number of structural and social conditions to be put in place that go beyond the capacities and responsibilities of individual doctors or individual technology designers. They refer to public healthcare, support by health and non-health professionals, participatory elements in health policy and in the development of new approaches – including technologies – to identify needs and interests of the target population. Most technologies on the market are still rather fixed in their options. As a result, their range of possibilities to adapt to the great variety of real personal interests and needs is limited. Therefore, a particularly broad implementation of participatory approaches is required when it comes to planning and designing such technologies. This requirement corresponds to current trends in bioethics to recognize perspectives of patients and their social surroundings, for example, by including their voices and experiences into bioethical discussions via qualitative, empirical, and participatory research (Raz & Schicktanz, 2016; Schicktanz et al., 2012).

Given the relevance of power interrelations in empowerment theory, it is important to critically reflect whether technology creates new dependencies. This power-critical perspective of empowerment goes beyond the mere interpretation of assistive technologies in terms of ‘enablement’ or ‘care’. Human power relations are – at least in theory – open for continuous renegotiation and reconfiguration. However, how much renegotiation is possible for an older user vis-a-vis a technological system? Notably, the term ‘empowerment’ is now used by some engineers and computer scientists to describe to what extent a technical agent (a robot, a machine) is in control of the world it can perceive (Salge & Polani, 2017). For them, empowerment is a concept to quantify the capacities of a robot to maintain or enhance its ability to act and control the environment in an operational, formalistic manner. This move of making machines more independent can, of course, collide with the goal of empowering patients who use such a technical assistive system, as autonomy of the system can come into conflict with the users’ autonomy. Another problematic turn in the use of ‘empowerment’ terminology is its occurrence in the recent emphasis of citizens’ responsibilities for their own health issues. This can nowadays be found in campaigns for the promotion of healthy behavior or in direct-to-consumer genetics. As long as these trends do not entail an extension of public participation or are strengthening chronic patients’ rights and capacities but rather indicate a shift to neoliberal reorganization of formerly public healthcare services, the usage of empowerment rhetoric should be criticized (Chiapperino & Tengland, 2015). Taken seriously, empowerment philosophy and the application of its practical conditions highlight particular requirements regarding technology development and engineering. Thus, the idea of empowerment demands that technological assistance is designed in a way that all older people can control the machine’s basic functions on their own. It also stresses the need for technical assistance that provides support for the interpretation of results or their explicability in a language that is accessible for the potential user groups. Hence, empowerment ultimately requires the participation of older users at all stages of technology development.

#### Privacy: protecting a personal way of life

In a similar way, the ethical concept of privacy in technology assisted eldercare also goes beyond the scope of traditional medical and nursing ethics considerations. In these contexts, privacy primarily refers to the professional virtue of doctors’ confidentiality and the protection of the physician-patient-privilege. It thus mainly concerns and covers personal information deliberately disclosed or revealed during an individual medical consultation and diagnosis or treatment process (Thompson, 1979).

In the context of technology-assisted eldercare, by contrast, privacy is not only about specific personal health-related information that the patient actively discloses to the doctor in a confidential consultation, or about the personal knowledge the doctor gains in the context of medical diagnosis or treatment. The effective implementation and functioning of technological assistance systems rather presupposes a more or less comprehensive and continuous monitoring of a person’s physical functions, movements and behaviors, as well as everyday life, on the basis of large amounts of real-life data. For example, sensors are used to control vital parameters, physiological functions, and behavioral patterns. Moreover, tracking devices collect data on an individual’s location and movements in space. Consequently, techno-ethical considerations make clear that such data-intensive technologies pose much more fundamental and far-reaching challenges to privacy than a single, clearly terminable doctor’s appointment (Kolkowska & Kajtazi, 2015). Thus, the user of a monitoring system does not decide to disclose a specific information but instead inevitably ‘emits’ data on a permanent basis. The idea that anyone could actually exercise an active and conscious control over these large and constant streams of data appears unrealistic and would effectively overburden any individual decision-maker. Accordingly, more generic concepts of informational self-determination and data sovereignty have to be developed, including technological privacy by design-solutions (Cavoukian et al., 2010).

Furthermore, apart from this *informational dimension*, technology-assisted eldercare also touches upon other aspects of privacy such as *decisional privacy*, that is, the right to make one’s own decisions about personal matters without interference by third parties (which is closely related to the idea of autonomy). However, while medical ethics safeguards decisional privacy through informed consent procedures that protect the patient’s self-determined decisions about treatment options, assistive technologies pose the even more fundamental question which decisions we are offered to make at all. Especially in the context of more autonomous learning systems, the objective usually is that the technology continuously and dynamically adjusts to (presumed) user needs and preferences that are determined by algorithms rather than being explicitly articulated. This way, many decisions may not even occur as such anymore but are rather automatically processed in the background, for example, when an ambient assisted living platform regulates the heating system in a household according to calculated optimal temperature values. The situation is further complicated by the problem of algorithmic opacity in learning systems that may not be able to provide an explanation of decisions made (Burrell, 2016). Under these circumstances, the challenge of decisional privacy is to decide about the range of decisions. This includes defining the scope of matters the individual would like to have submitted for active and explicit decision – and the range of other things simply left to the control of the automated system. The risk of (progressive) technological disempowerment and incapacitation calls for ethical and technological approaches to decisional privacy that can keep the user ‘in the loop’ (Morte-Ferrer et al., 2020).

Eventually, the continued presence of assistive technologies in eldercare also affects the *topological dimension* of privacy. The respective technologies do not simply enter into – and afterwards leave – a private sphere from the outside like a doctor on a house call. They usually become an integrative part of the users’ closer life world and living environments. For example, technological devices like a companion robot or a smart home system can hardly be conceptualized as an external intruder. In fact, they are often deliberately designed to be ‘unobtrusive’. Ambient technologies are virtually defined by the systematic reduction of visibility and user interfaces. Yet such systems merge into the private homes and living arrangements of the users and thus may fundamentally modify the way older people and their relatives perceive themselves and feel and behave within their domestic spaces and private lives. This happens, for example, by tacitly monitoring and modulating daily routines and habits of food intake, personal hygiene, or leisure activities. When such technological systems are even designed to interpret user’s inner mental or emotional states in order to react to them, this can raise fundamental questions of selfhood, authenticity, and trust-building in personal relationships that transcend the doctor/care taker-patient relationship (Elder, 2016). A further, particularly important aspect is that such technologies point beyond traditional individualistic notions of privacy and instead concern social settings and arrangements that call for relational conceptions of topological privacy.

All in all, these expansions of the concept of privacy in the context of technology-assisted eldercare lift the pertinent ethical reflection to a whole new level. They not only raise issues of data protection that require technological safeguards and solutions and thus call upon the professional competences and responsibilities of engineers and computer scientists. They also highlight the intricate ethical issues of decision-making and integrity of the lifeworld involved in human-machine-interaction and socio-technical systems. In line with pertinent philosophical discussions of privacy, they thus ultimately touch upon fundamental theoretical questions regarding the constitutive preconditions of autonomy, (human) agency, personal identity and (social) relations (Parent, 1983).

### Reconsidering care and harm by technical assistance: issues of quality of life and liability

Issues of care in assistive technologies for eldercare also point beyond the principlist perspective of medical and nursing ethics, where the concept of care comprises primarily the principles of beneficence and non-maleficence. Doctors and nurses have the professional responsibility to protect and promote the well-being of their patients and to avoid harm. In this context, ideas of well-being and harm and the corresponding beneficent and non-maleficent professional practices are mainly viewed through the lens of health and healing (Pellegrino, 1988).

The ethical discussion of care in the context of assistive technologies in eldercare, however, goes beyond this point. In particular, it has to include genuinely techno-ethical issues of safety and security of technical devices and systems, as well as questions of moral responsibility and legal liability in case of accidents and malfunctions. Furthermore, it also becomes relevant to reflect on the normative assumptions of what ‘social’ vs. ‘technical’ assistance implies for the quality of care. Thus, the question is whether, and to what degree, social assistance is better than technical assistance, or how both can be combined in order to promote the well-being of users.

Technical assistance in (elder) care expands the viewpoint on what can be considered as consequential perspectives of well-being or harm. Especially the fact that technical systems can add to the spectrum of potential harms should be examined much more closely. In human-human care interactions, harms as side-effects (not violence as a co-phenomenon in care situations, e.g., as a sign of overburdening or hidden aggressions between caretaker and patient) are often conceptualized in terms of ‘bad’ and insufficient care. However, technical assistance can cause harm to a person in very different ways. Robotic systems that involve robot arms or moving parts can, for example, physically injure a person. This is a technical risk when robot arms or mobile parts of a robot have various degrees of freedom to move and the technical control system is AI-steered so that the movement may not always be predictable for the patient or caretaker. Similarly, so-called smart beds or body lifters might get stuck in a particular position that can be harmful to the patient’s body. This is a technical risk to be considered, as technical errors can simply happen, for example, under power outage. In such cases, the question of human control over the technical system becomes most relevant.

Furthermore, the issue of liability in many cases remains unsolved. Whether engineers and programmers are also liable in such cases is not sufficiently clarified. A proposal for governance of such technical developments will help increase trust in and reduce potential harms of such AI robots (Winfield & Jirotka, 2018). However, the process of governance must result in clear and transparent regulations of liabilities in cases of technical errors, as it is unlikely that they will never occur – a lesson the history of technology has taught us (see, e.g., Schlager, 1994). The professional responsibilities of caretakers or doctors for well-being and non-harming therefore have to be expanded by the engineer’s ethical norms of risk reduction or even precautionary risk management. This in turn can, however, create new dilemmas, especially where the traditional engineer’s ethics based on the first law of robotics “do not harm” collides with subjective valuing of technical assistance by the patients themselves. Even if such cases are hopefully rare, one should anticipate these potential problems of technical assistance in advance and clarify responsibilities.

The categories of non-harming and well-being are also dependent on subjective interpretations of quality of life. The increasing interest in patient reported outcomes takes this subjectivity into account in linking well-being with empowerment (McAllister et al., 2012). Care ethics often tend to prioritize human care over technical assistance. They highlight the social aspects of empathy, closeness, and intimacy and present technical care as a purely instrumental form of care that cannot cover such social features. However, qualitative studies with patients with serious physical disabilities indicate that technical care is actually welcomed in some areas of bodily hygiene (Schicktanz et al., 2015). Technical support that could make human help unnecessary when it comes to toilet or shower use thus would not only be valued from the point of view of self-determination, but also from the perspective of subjective well-being. Here, experiences of intimacy and privacy are linked to subjective quality of life.

All in all, these specifications to the principle of care in the context of assistive technologies for eldercare expand the scope of ethical reasoning in several directions. They raise issues of malfunctioning and operational safety of care technologies and the resulting harms, as well as questions regarding the specificities of genuinely human care compared to technological assistance. This way, they also point towards much more complicated issues of well-being, social responsibility and legal liability of engineers and computer scientists. This ultimately raises fundamental praxeological and moral philosophical questions regarding the meaning, scope and attribution of accountability, responsibility, and liability.

### Justice: integrating welfare and sustainability

In the context of medical ethics, the principle of justice originally referred to the doctor’s professional duty to treat all patients with the same care and respect. It thus basically involved the precept of equal treatment and the prohibition of any discrimination, for example, on the basis of age. Only later was this principle expanded to more general issues of distributive justice in the context of resource allocation in the healthcare system (Beauchamp & Childress, 2013).

In the context of technology-assisted eldercare, this normative principle of justice must also be discussed from a broadened perspective. The professional ethics of doctors and other healthcare workers was traditionally based on a strong humanitarian ethos of altruism and beneficence and a certain distance to the commercial principles of the market sphere (Pellegrino, 1988). Healthcare is widely regarded as a public good, the distribution of which should not be left to the market alone (Daniels, 1985, 2008). There have been long controversies about the inappropriateness of a commercialization of healthcare, the profit-oriented logic of the development and allocation of drugs or medical devices, and the problem of market failure in the healthcare system (Arnold, 2009; Weber, 2006).

By contrast, technology development frequently originates from the economic context of commerce and industry and is intrinsically driven by commercial interests. It involves an economic investment that results in patents and products that promise economic exploitation and profit. Consequently, the professional practice of engineering and computer sciences does not preclude these economic motives and does not necessarily involve a systematic critical reflection on their ethical implications. Indeed, in the past, engineers have frequently been singled out as a prominent target for the philosophical critique of commodification and purely instrumental reason that could serve any arbitrary interests and purposes (Schecter, 2010). Although this line of critique is surely rather blunt and exaggerated, it raises the fundamental techno-ethical question of an ethos, an internal morality of engineering and information science that could justify resistance and limits to processes of economization or at least constitute a substantial counterweight (Harris, 2008). As a matter of fact, questions of just access to assistive technologies have only just begun to be discussed in a more systematic way (Durocher et al., 2019).

Developing assistive technologies for eldercare implies different levels of economization, with costs not only for production and distribution, but possibly with even higher costs for long-term management and sustainable functioning. Furthermore, these economic dimensions also require an in-depth consideration of how technological care goes hand in hand with a particular version of neoliberal economization that shifts care responsibilities away from the state and towards the individual and the family (Folbre, 2006; Kenner, 2008). Indeed, the political debate of healthcare technologies in eldercare is largely dominated by two divergent rhetorical paradigms (Neven & Peine, 2017). On the one hand, there is the deficit-oriented discourse of austerity and rationing scarce resources in the face of demographic aging that portrays technology as a cost-efficient surrogate and replacement in the face of shortage of human care workers and precarious or shrinking social systems. On the other hand, there is the promissory and prestigious discourse of dynamic, cutting-edge technology development and eldercare as a highly competitive and profitable future market. Both paradigms are primarily oriented towards economic aspects of technology development and do not yet sufficiently reflect the ethical implications for eldercare. The first one conveys negative, deficit-oriented stereotypes of aging and older people in terms of individual decline and apocalyptic demography, while the second promotes a problematic view of technological innovations that easily underestimates risks for the safety and well-being of individual users as well as problematic societal consequences (Neven & Peine, 2017). A critical reflection of this larger context of socio-economic interests and framework conditions of technology development for eldercare is definitely needed in order not to take the implementation of assistive technologies simply as a given fact or an inevitable necessity.

Finally, compared to many medical interventions, the broad implementation of technological innovations can have rather far-reaching consequences for the working situation of and labor market for healthcare professionals, the structure of social relations, and the protection of the ecological environment. Thus, in this context, questions of justice ultimately point beyond matters regarding equal treatment and just distribution of resources. Societies not only have to decide what financial investments they are prepared to make into the technological support and improvement of eldercare. A long-term perspective also has to consider large-scale economic consequences as well as sustainability and responsibility regarding future generations. Thus, ecological consequences due to increased use of energy and scarce resources may become relevant and pose the question of how the large-scale production and operation of such technologies can be achieved with renewable energy sources and low waste.

In sum, these specifications of the principle of justice in the context of technology-assisted eldercare expand the theoretical framework of ethical reflection in several ways. They point beyond the traditional professional ethos of physicians and nurses and raise questions of an ethics of engineering and computer sciences. In doing so, especially the social and economic embeddedness and implications of care technologies come to the fore. They ultimately touch upon fundamental questions of close social relations, intergenerational responsibilities and even long-term ecological perspectives regarding the environment and sustainability.

## Discussion and outlook

The emergence of new assistive technologies will foreseeably change many practices and conditions of care for older people. This does not necessarily mean that established theoretical frameworks and normative principles of ethical reflection in medicine and healthcare become obsolete. Nevertheless, it appears important to expand the ethical perspective in at least three respects.

First, ethical reflection in the context of assistive technologies in eldercare needs to broaden the scope beyond the domain of traditional medical ethics in order to consider the professional ethos of engineering and computer science. This introduces new techno-ethical perspectives and concerns into the debate, for example, aspects of technological empowerment and privacy, safety, responsibility and liability, or technological economization and commercialism. It thus enriches professional ethical guidance in the context of medicine and healthcare and opens a new field of inter- and multi-professional cooperation between medical ethics and ethics of technology.

Second, the introduction of assistive technologies into eldercare touches upon an irreducibly social dimension of ethical reflection. It transcends the theoretical and methodological individualism of principlist approaches to medical ethics and their focus on conflicts arising in the personal doctor-patient-interaction in concrete situations. Instead, a variety of different stakeholders are concerned and therefore need to be considered in the ethical debate, for example, patients, relatives, professional caregivers and care homes as well as developers and producers with their respective interests and concerns. Thus, more complex social constellations, structural conditions, and institutional frameworks come to the fore. As a consequence, it also seems much more common to view issues of technology development and implementation as genuinely political matters that call for public participation and deliberation. Thus, since many technological systems are still in their infancy and detailed user experiences are rare, it becomes more important to include the real addressees into technology development and the pertinent public and political debates.

Finally, the emergence of human-machine-interaction and cyber-physical systems in eldercare also raises fundamental ontological, anthropological, praxeological, and social philosophical questions. Thus, it challenges traditional understandings of subjectivity, agency, autonomy, accountability, and social relations that are usually tacitly presupposed in applied medical ethics and nursing ethics discussions. This means that the principlist abstinence from matters of (moral) philosophical foundation and its pragmatic self-restriction to a set of middle-range ethical principles is not sufficient in this area. In order to conceptualize the moral problems and conflicts occurring in the context of assistive technologies for eldercare in an adequate manner, ethical reflection has to engage in a discussion of the underlying theoretical concepts and assumptions.

Against this backdrop, rethinking the ethical framing of technology-assisted eldercare appears necessary. While it would be counterproductive to reject bioethical principlism in healthcare altogether, it falls short in the context of assistive technologies for eldercare. As we have argued, its traditional medical focus needs to be expanded in order to include professional perspectives and ethical principles from engineering and computer science and engage in interdisciplinary and interprofessional exchange. The social and political dimensions of moral conflicts have to be taken into account, which calls for public participation and deliberation in ethical controversies. Fundamental questions of agency, autonomy, accountability, and social relations arise and require the discussion of moral philosophical foundations. The further systematic consideration of these aspects will enrich and strengthen the ethical reflection of assistive technologies in eldercare.
